# Evolution and precision trends of pancreaticojejunostomy in pancreaticoduodenectomy

**DOI:** 10.3389/fonc.2026.1818437

**Published:** 2026-07-22

**Authors:** Jianrong Cheng, Di Zhang, Chunxia Wang, Feng Cui, Guodong Wang, Xin Zhao, Kaidong Xu, Xingzhong Fang, Jinhong Chen

**Affiliations:** 1 Department of General Surgery, The First People's Hospital of Lanzhou City, Lanzhou, Gansu, China; 2 Department of General Surgery, Affiliated Hospital of Gansu University of Chinese Medicine, Lanzhou, Gansu, China; 3 Department of General Surgery, The Second People's Hospital of Lanzhou City, Lanzhou, Gansu, China; 4 The First Clinical Medical College, Gansu University of Chinese Medicine, Lanzhou, Gansu, China; 5 Department of General Surgery, The Second People's Hospital of Dingxi, Dingxi, Gansu, China

**Keywords:** pancreatic cancer, pancreaticoduodenectomy, pancreaticojejunostomy, postoperative pancreatic fistula, surgery

## Abstract

Pancreatic cancer is a highly malignant tumor associated with a generally poor prognosis. Pancreaticoduodenectomy remains the only potentially curative surgical treatment for pancreatic head and periampullary cancers. As the core step of Pancreaticoduodenectomy, pancreaticojejunostomy is directly associated with postoperative complications, particularly postoperative pancreatic fistula (POPF), which significantly impacts patient outcomes. Despite continuous advancements in surgical techniques, the risk of POPF remains high. Its occurrence is closely related to patient baseline status, extent of tumor invasion, pancreatic texture, main pancreatic duct diameter, and the choice of anastomotic technique. The selection of anastomotic technique should be individualized based on the local anatomical characteristics of the pancreas, overall patient condition, and surgeon experience. However, no universally accepted standard technique has yet been established internationally. To this end, this article systematically reviews the developmental history of pancreaticojejunostomy techniques and explores their evolving trend toward precision, aiming to provide a theoretical basis and practical reference for surgeons to perform precise and safe pancreaticojejunostomy during Pancreaticoduodenectomy.

## 1 Introduction

Pancreatic cancer(PC), one of the malignancies with the poorest prognosis, poses a substantial challenge to global public health systems. According to cancer epidemiology statistics, there were approximately 510,000 new cases of PC worldwide in 2022, ranking it 12th among all malignancies, and approximately 470,000 deaths, ranking it 6th (1). The disease has an insidious onset, with the majority of patients diagnosed at advanced stages, severely impacting quality of life, and the 5-year overall survival rate is only 5%–10% (2). Pancreatic head and periampullary cancers account for approximately 60%–70% of pancreatic malignancies, and pancreaticoduodenectomy (PD) remains the only potentially curative treatment.

PD involves complex anatomy and requires multiple gastrointestinal reconstructions, including pancreaticojejunostomy, hepaticojejunostomy, and gastrojejunostomy, as well as systematic lymph node dissection. Among these, pancreaticojejunostomy (PJ) is the core step of the procedure (3). The postoperative complication rate of PD ranges from 30% to 50%, with common complications including pancreatic fistula, biliary fistula, delayed gastric emptying, biliary stricture, hemorrhage, and infection (4). Among all complications, postoperative pancreatic fistula (POPF) is particularly prominent, with an incidence of 10%–35% (5). POPF can lead to secondary intra-abdominal hemorrhage, infection, and even multiple organ failure, and is an important risk factor for patient mortality.

PJ is widely regarded as the most technically demanding step with the highest risk of POPF, as well as the focus where anastomotic techniques have evolved most frequently and continue to stimulate surgical exploration. Nevertheless, no universally accepted “gold standard” PJ technique exists, and different anastomotic methods each have their own technical characteristics and indications. Therefore, this article aims to review the development and technical evolution of PJ techniques, summarize the key operative points, indications, and clinical outcomes of current mainstream methods, and provide a reference for surgeons in selecting and optimizing anastomotic strategies in clinical practice.

## 2 Literature search and selection strategy

To delineate the evolutionary trends of pancreaticoduodenectomy and pancreaticojejunostomy techniques, we searched the PubMed and CNKI databases from their inception to December 2025 using the following terms and their synonyms: pancreaticoduodenectomy, pancreaticojejunostomy, postoperative pancreatic fistula, pancreatic cancer, and periampullary cancer. Included study types comprised randomized controlled trials, cohort studies, systematic reviews, and meta-analyses, with content focusing on surgical techniques, imaging assessment, treatment strategies, clinical outcomes, or complications. Exclusion criteria included studies not related to pancreaticoduodenectomy (e.g., other types of pancreatic surgery) and conference abstracts lacking complete data. Literature screening was independently conducted by two researchers using EndNote 20 for reference management. Studies were screened sequentially by title, abstract, and full text, and any disagreements were resolved through discussion with a third researcher. Core information from the included studies (authors, year, study type, sample size, methods, outcomes, and conclusions) was extracted using a standardized form, and cross-checking was performed to ensure data completeness and accuracy.

## 3 Historical evolution of pancreaticoduodenectomy

PJ is a core step in reconstructing the continuity between the pancreatic remnant and the digestive tract. The choice of anastomotic technique and the quality of the anastomosis are independent risk factors influencing the incidence of severe complications, particularly POPF, following PD. The evolution of this anastomotic technique has undergone continuous refinement from the era of traditional open surgery to the minimally invasive age. During the classic open PD era, surgeons established fundamental anastomotic principles and developed multiple techniques for PJ, including duct-to-mucosa anastomosis, invagination anastomosis, and various modified reinforcement methods (6). The core objectives of these techniques have consistently focused on achieving effective pancreatic juice drainage, ensuring anastomotic seal integrity, and maintaining adequate blood perfusion to minimize the risk of POPF. With the introduction of laparoscopic and robot-assisted surgery, PD has entered the minimally invasive era, while also presenting new challenges (7, 8). Laparoscopic surgery, facilitated by magnified visualization, has enhanced the refinement of anastomotic construction; however, it also faces difficulties such as restricted maneuvering angles and increased suturing complexity. The robotic surgical system, leveraging its three-dimensional high-definition visualization and the flexible advantages of wristed instruments, enables more precise and controllable completion of complex pancreatic duct suturing and tissue approximation within confined spaces, thereby further advancing PJ techniques toward minimally invasive and standardized development. Nevertheless, its clinical application has not yet become widespread (9, 10).Currently, multiple PJ techniques exist in clinical practice, with selection and implementation strategies requiring individualized decision-making based on key factors including pancreatic texture, pancreatic duct diameter, surgeon experience, and institutional resources. The following sections will systematically review the evolutionary development of several mainstream anastomotic techniques.

## 4 Different techniques of pancreaticojejunostomy

### 4.1 Classification based on anastomotic site

#### 4.1.1 Pancreaticojejunostomy

PJ involves anastomosing the pancreatic remnant to the jejunum. As a traditional pancreaticoenteric anastomosis technique, it is currently the most commonly used reconstruction method in PD. This procedure typically employs Roux-en-Y reconstruction, in which a segment of the jejunal limb is selected for anastomosis with the pancreatic remnant, thereby establishing an independent drainage pathway for pancreatic juice directly into the jejunum. This configuration relatively separates pancreatic juice from bile and gastric contents, helping to reduce local anastomotic injury caused by pancreatic enzyme activation. Additionally, the jejunal limb has a rich blood supply (primarily from branches of the superior mesenteric artery), providing strong healing capacity at the anastomosis and helping to reduce ischemia-related complications (11). Multiple randomized controlled trials and meta-analyses have demonstrated that compared with other anastomotic techniques, PJ is associated with lower incidences of long-term complications including anastomotic stricture, chronic pancreatitis, and steatorrhea (12, 13), with particularly favorable outcomes in patients with firm pancreatic texture and dilated pancreatic ducts (14). By virtue of its physiological drainage pathway, favorable POPF risk profile, and robust vascular supply, PJ has become the preferred technique for pancreatic duct reconstruction following PD. Although this technique demands considerable surgical expertise, when performed by experienced surgeons, PJ provides safer and more stable postoperative outcomes (**Table 1**).

**Table 1:** Comparison of pancreaticoenteric anastomotic techniques classified by anastomotic site.

Anastomotic type	Technique	Advantages	Disadvantages	Indications
Pancreaticojejunostomy	Anastomosis of the pancreatic remnant to the jejunum	1. Independent pancreatic juice drainage pathway, relatively separating pancreatic juice from bile and gastric contents, reducing local injury. 2. Rich jejunal blood supply with strong healing capacity. 3. Lower incidence of long-term complications (anastomotic stricture, chronic pancreatitis, steatorrhea).	Technically demanding	Particularly favorable in patients with firm pancreatic texture and dilated pancreatic ducts; currently the most commonly used reconstruction method.
Pancreaticogastrostomy	Direct anastomosis of the pancreatic remnant to the posterior or anterior gastric wall; pancreatic juice is drained into the gastric lumen	1. Technically simpler. 2. Excellent anastomotic blood supply and healing capacity. 3. Gastric acid inhibits pancreatic enzyme activity, reducing autodigestion.	1. Increased risk of postoperative hemorrhage (reduced with double-layer suturing). 2. Possible delayed gastric emptying and gastric content reflux.	Patients with soft pancreatic texture, small main pancreatic duct (<3 mm), or difficult duct identification.
Pancreaticoduodenostomy	Direct anastomosis of the pancreatic remnant to the duodenal stump	1. Restores physiological pancreatic juice drainage. 2. Preserves anatomical continuity and physiological function of the digestive tract.	1. Applicable only in duodenum-preserving pancreatic head resection.	Duodenum-preserving pancreatic head resection (e.g., for benign or low-grade malignant tumors).

#### 4.1.2 Pancreaticogastrostomy

Pancreaticogastrostomy (PG) is an important alternative technique for pancreatic duct reconstruction in PD. The core procedure involves direct anastomosis of the pancreatic remnant to the posterior or anterior gastric wall, diverting pancreatic juice into the gastric cavity. The principal advantages of this technique include relative technical simplicity, robust anastomotic blood supply, and gastric acid-mediated inhibition of pancreatic enzyme activity, which reduces the risk of anastomotic autodigestion caused by enzyme activation (15). Current evidence indicates that overall POPF rates following PG are comparable to those of PJ (16). However, some single-center randomized controlled trials have suggested that PG may be significantly superior to PJ with respect to POPF incidence, intra-abdominal abscess formation, and hospital stay duration, while no significant differences in mortality or other complications have been observed between the two approaches (17, 18). Of note, PG may be associated with a significantly increased risk of postoperative hemorrhage, although this risk can be mitigated by employing double-layer anastomotic techniques (19). Furthermore, this technique may potentially induce long-term alterations in gastric emptying dynamics, gastric content reflux, and possible effects of pancreatic enzymes on the gastric mucosa. Long-term outcome data for PG remain less systematic and comprehensive compared with those for PJ. Existing studies have identified soft pancreatic texture as an independent risk factor for POPF. Consequently, in patients with soft pancreatic parenchyma and a small main pancreatic duct (<3 mm) or difficult duct identification, PG may offer distinct clinical advantages (20–22).

#### 4.1.3 Pancreaticoduodenostomy

Pancreaticoduodenostomy (PDS) is a relatively infrequently used pancreatic duct reconstruction technique. The main procedure involves direct anastomosis of the pancreatic remnant to the duodenal stump under conditions of preserved duodenal integrity, thereby restoring physiological pancreatic juice drainage. In classic PD, the duodenum is typically resected entirely to achieve adequate tumor-free margins; therefore, traditional PDS is essentially no longer employed following standard PD. However, in duodenum-preserving pancreatic head resections, PDS retains certain clinical applicability (23, 24). This technique permits pancreatic juice drainage through the duodenum, thereby preserving both the anatomical continuity and physiological function of the digestive tract.

### 4.2 Classification based on anastomotic technique

#### 4.2.1 Duct-to-mucosa pancreaticojejunostomy

Duct-to-mucosa pancreaticojejunostomy is a technique that involves direct anastomosis between the main pancreatic duct and the jejunal mucosa, followed by suture approximation of the pancreatic parenchyma to the jejunal seromuscular layer. This method establishes a continuous pancreatic duct-jejunal mucosal conduit, which facilitates long-term anastomotic patency and preserves exocrine pancreatic function, while the jejunal serosal coverage over the pancreatic remnant provides protection to the pancreatic parenchyma. Among these approaches, the Blumgart anastomosis represents the most widely utilized and classic duct-to-mucosa technique, with multiple studies confirming its safety, reliability, and efficacy in reducing POPF rates (25–27). However, duct-to-mucosa suturing demands considerable surgical precision and may present challenges for less experienced surgeons. In response, Hong et al. (28) proposed the “fistula tract healing” theory and introduced technical innovations: placing a drainage tube of appropriate caliber into the main pancreatic duct and extending it into the intestinal lumen to create a long-term indwelling “artificial fistula tract.” This guides gradual approximation and healing between the pancreatic duct and jejunal mucosa, allowing time for adhesive healing between the pancreatic remnant and jejunal seromuscular layer. A study involving 1,033 patients demonstrated that this anastomotic technique achieved a POPF rate of 5.9% (grade B/C), superior to traditional techniques, with a median PJ time reduced to 24 minutes, thereby enhancing overall efficiency in laparoscopic procedures (29). This approach not only facilitates shortening of the learning curve but also represents a preferred option for patients with identifiable ducts across varying pancreatic duct diameters and parenchymal textures (**Table 2**).

**Table 2:** Comparison of pancreaticoenteric anastomotic techniques classified by anastomotic technique.

Anastomotic type	Technique	Advantages	Disadvantages	Indications
Duct-to-mucosa pancreaticojejunostomy	Direct anastomosis between the main pancreatic duct and the jejunal mucosa	1. Maintains long-term anastomotic patency and preserves exocrine pancreatic function. 2. Jejunal serosal coverage provides protection to the pancreatic remnant.	1. Technically demanding, requiring substantial operator experience. 2. Challenging in patients with soft pancreatic texture and small pancreatic duct diameter.	Patients with identifiable pancreatic duct.
Invagination pancreaticojejunostomy	Invagination of the pancreatic remnant into the jejunal stump or side wall to achieve anastomotic contact through encapsulation	1. Large contact area and high mechanical strength. 2. Particularly suitable for patients with soft pancreatic texture and small pancreatic ducts. 3. Reduced technical difficulty.	1. Risks of remnant hemorrhage, pancreatic duct stricture, and secondary pancreatitis. 2. Difficulty in invagination when the pancreatic remnant is excessively large.	Patients with soft pancreatic texture and small pancreatic ducts.

#### 4.2.2 Invagination pancreaticojejunostomy

Invagination pancreaticojejunostomy primarily encompasses two approaches: end-to-end invagination and end-to-side invagination. End-to-end invagination anastomosis was widely employed in open surgery, with the core technique involving partial invagination of the pancreatic remnant into the jejunal stump lumen, achieving maximal contact area and mechanical strength through “encapsulation,” particularly suitable for patients with soft pancreatic texture and small pancreatic ducts (30). However, when the pancreatic remnant is excessively large, invagination manipulation often proves technically challenging. End-to-side invagination anastomosis involves creating an incision on the jejunal side wall slightly larger than the pancreatic remnant, inserting the mobilized pancreatic remnant into the intestinal lumen, and completing the anastomosis with purse-string sutures or binding fixation (31, 32). Nevertheless, invagination anastomosis requires extensive mobilization of the pancreatic remnant with direct exposure of the transected surface to the intestinal lumen, posing risks of intestinal fluid erosion leading to remnant hemorrhage, pancreatic duct stricture, and even secondary pancreatitis. In view of these concerns, Chen et al. (33) proposed a semi-invagination technique that significantly reduces the aforementioned complications. This technique initially employs two U-shaped through-and-through sutures to fix the pancreas and jejunum, places a stent tube into the main pancreatic duct and introduces it into the intestinal lumen, and subsequently embeds the pancreatic remnant through interrupted or continuous U-shaped full-thickness sutures. This method does not depend on diameter matching between the pancreatic remnant and intestinal lumen, nor does it require suturing between the pancreatic duct and jejunal mucosa, thereby reducing surgical difficulty while avoiding ductal stricture or occlusion resulting from pancreatic duct suturing, and minimizing postoperative hemorrhage risk associated with digestive juice contact with the pancreatic transection surface (34).

## 5 Precision trends in pancreaticojejunostomy

As the core component of PD, the precision-oriented development of PJ has become an important trend in recent years. Driven by the concept of precision surgery, this trend is mainly reflected in the following aspects: individualized selection of anastomotic techniques, introduction of minimally invasive and robot-assisted surgical platforms, imaging-guided intraoperative decision-making, application of novel biomaterials, and systematized perioperative and postoperative care. It should be noted that prevention of POPF does not depend solely on the anastomotic technique itself, but involves comprehensive management of multiple factors, including pancreatic texture, pancreatic duct diameter, drainage strategies, biochemical monitoring parameters, the use of somatostatin and its analogues, nutritional support, implementation of enhanced recovery after surgery (ERAS) pathways, and the establishment of early identification and salvage treatment strategies.

### 5.1 Individualized selection of pancreaticojejunostomy techniques

As surgical practice advances toward precision medicine, traditional techniques no longer adequately address the diverse needs of different patients. Individualized selection of PJ techniques represents a core practice of the precision surgery concept, aiming to transcend the limitations of uniform conventional approaches through comprehensive decision-making that integrates multiple factors including pancreatic tissue texture, pancreatic juice secretion characteristics, patient baseline status, surgical scenario, and operator experience. This decision-making process extends beyond selection of anastomotic type to encompass systematic integration and combinatorial application of technical elements including suturing methods and number of suture layers. Regarding suturing techniques, selection should align with technical characteristics: interrupted suturing, through independent fixation of each stitch, prevents failure of the entire anastomosis due to a single point of failure, thereby improving anastomotic safety; continuous suturing facilitates uniform tension distribution, reduces dead space and tissue cutting, proving particularly suitable for laparoscopic procedures (35); U-shaped sutures increase tissue-bearing thickness through transverse penetration of pancreatic parenchyma, potentially reducing remnant hemorrhage and leakage from small pancreatic ducts (36); through-and-through suturing offers advantages of fewer stitches and enhanced stability, improving operative efficiency while avoiding tissue tearing (37). On this basis, Peng et al. (38) further proposed differentiated selection based on pancreatic duct diameter: interrupted sutures for ducts ≤2 mm, continuous sutures for ducts ≥4 mm, and flexible intraoperative decision-making for ducts between 2–4 mm. Concerning suture layers, decisions should integrate pancreatic status with reconstruction objectives: single-layer anastomosis requires relatively shorter operative time, offers technical simplicity, and minimizes suture-related tissue injury, rendering it suitable for cases with firm pancreatic texture, dilated pancreatic ducts, or when stable mucosa-to-mucosa apposition can be achieved intraoperatively (39–41). Double-layer or multilayer suturing theoretically enhances seal integrity and stability, particularly for soft pancreata, albeit with increased technical complexity and potential for additional tissue trauma (42). Therefore, individualized PJ does not entail mechanical application of any single technique, but rather represents a strategic selection based on a profound understanding of various technical elements; incorporating patient-specific anatomical and pathological features into the reconstruction decision renders it more clinically convincing.

The connotation of precision surgery extends far beyond mere operative refinement. For example, in patients with a history of Roux-en-Y gastric bypass, the altered gastrointestinal anatomy presents challenges for reconstruction after PD. The authors’ successful management by preserving the remnant stomach and the original biliopancreatic limb strongly supports the core argument of this article: no single reconstruction method is suitable for all patients (43). Intraoperatively, flexibility and precision should be organically integrated to construct a stable and patent PJ, thereby effectively enhancing surgical safety and long-term patient outcomes.

### 5.2 Application of minimally invasive and robot-assisted surgery

With technological advances and increasing patient demand, laparoscopic pancreaticoduodenectomy (LPD) has gradually become a mainstream direction in the field of clinical PD. However, it must be emphasized that open PD remains the standard procedure, particularly for tumors with vascular involvement, reoperative cases, and low-volume centers. Due to its steep learning curve and high technical demands for PJ, LPD can currently be performed safely only in high-volume centers by experienced surgeons. Despite these limitations, recent high-quality evidence from high-volume centers supports its clinical value. Studies have shown that LPD achieves comparable rates of short-term severe complications and mortality to open PD, while offering significant advantages in shortened hospital stays and reduced intraoperative blood loss (44–46).

From an operational perspective, minimally invasive surgery not only offers clinical benefits of reduced trauma and faster recovery, but its magnified visualization and fine manipulation capabilities also help to better preserve pancreatic remnant blood supply and reduce anastomotic tension during PJ (47). Although the robotic surgical system, with its flexible instrument arms, high-definition three-dimensional visualization, depth perception, and ergonomic advantages, can partially overcome the technical difficulties of laparoscopy, its clinical application is not yet widespread (48). Adachi et al., through comparative investigation, demonstrated that robotic pancreaticoduodenectomy offers significant advantages in reducing intraoperative blood loss and shortening hospital stay, with the PJ component benefiting from precise robotic suturing that markedly improves local anastomotic accuracy (49). Additionally, robot-assisted surgery maintains consistent tension and fine needle spacing during suturing, which is crucial for ensuring pancreatic juice drainage and reducing POPF risk. Numerous studies comparing conventional laparoscopic versus robotic surgery regarding anastomotic outcomes have generally indicated superior performance of robotic technology in terms of blood loss and recovery time (9, 50, 51).

### 5.3 Imaging-guided precision localization

Modern surgery increasingly emphasizes preoperative precision localization, with image guidance technology constituting an essential component. Utilizing thin-slice contrast-enhanced CT, magnetic resonance imaging, and endoscopic ultrasonography combined with three-dimensional visualization and vascular reconstruction, the fine anatomical structures of the pancreas, tumor, and surrounding vasculature can be clearly delineated, allowing prediction of pancreatic duct trajectory, vascular anomalies, and pancreatic remnant status, thereby enhancing surgical safety. Clinical data demonstrate significantly reduced incidences of postoperative complications including POPF, hemorrhage, and infection with image-guided techniques compared to conventional approaches (52).

Research by Ghimire and Maharjan’s teams demonstrated that intraoperative indocyanine green (ICG) fluorescence imaging effectively assesses pancreatic remnant vascular perfusion during PD, enabling intraoperative adjustment of unevenly vascularized regions to optimize anastomotic quality and reduce POPF risk (53, 54). However, current studies have certain limitations, and future multicenter, prospective, and randomized controlled trials are urgently needed to further validate its clinical value. Furthermore, three-dimensional reconstruction based on CT data enables surgical simulation, facilitating preoperative virtual operative training that enhances technical proficiency and surgical safety, thereby reducing postoperative complications of PJ.

### 5.4 Perioperative comprehensive management for POPF prevention

Prevention of clinically relevant POPF after PD depends not only on continuous refinement of anastomotic techniques, but also on systematic management strategies throughout the perioperative period. Among these, prehabilitation, nutritional support, and frailty assessment have gradually become core components of the comprehensive PD management pathway. Preliminary studies have shown that patient-led, unsupervised outpatient home-based prehabilitation, although feasible, did not significantly improve postoperative clinical outcomes. Structured, multidisciplinary, individualized prehabilitation strategies targeting objective indicators (e.g., grip strength, cardiopulmonary function) may represent an important direction for future research (55).

Preoperatively, key factors including pancreatic texture, main pancreatic duct diameter, body mass index, and nutritional status should be comprehensively assessed to construct individualized risk prediction models. For patients with severe jaundice or hypoalbuminemia, timely biliary drainage and nutritional support should be implemented. Intraoperatively, meticulous management of the pancreatic remnant is required, with individualized selection of anastomotic techniques based on pancreatic texture and duct diameter. Routine placement of an intra-abdominal drain near the anastomosis facilitates adequate drainage. Postoperatively, dynamic monitoring of drain fluid amylase levels and infection indicators should be performed, with maintenance of effective drainage and negative-pressure irrigation when necessary. Early enteral nutrition should be prioritized, ERAS pathways followed, and somatostatin analogues used appropriately. Current evidence indicates that prophylactic use of somatostatin analogues (SA) reduces POPF rates and overall complication rates, but has not been shown to reduce mortality (56). For patients with biochemical leak or grade B POPF, a comprehensive management strategy centered on conservative drainage and nutritional support is recommended. If progression to grade C POPF or other severe complications occurs, timely reoperation should be undertaken. Through the above perioperative comprehensive management, the incidence and severity of POPF can be minimized.

### 5.5 Systematized postoperative care strategies

Postoperative care following PJ in PD should target POPF reduction and enhanced recovery, encompassing key components including postoperative monitoring, drain management, nutritional and metabolic support, pain control, early mobilization and rehabilitation, and discharge planning with follow-up. Postoperative monitoring should integrate vital signs, abdominal findings, drain output characteristics and volume, and amylase levels, combined with imaging assessment to enable early identification and stratified intervention (57). Drain management emphasizes individualized strategies and timely removal assessment to minimize infection risk while maintaining anastomotic stability (58). Nutritional and metabolic aspects emphasize early postoperative enteral nutrition, glycemic control, and parenteral nutrition when necessary to ensure adequate energy and protein supply for healing (59). Pain management employs multimodal analgesia integrated with ERAS principles to alleviate discomfort, reduce opioid dependence, and promote respiratory function and mobility recovery (60). Early mobilization and rehabilitation protocols encourage early ambulation, respiratory exercises, and physical therapy to reduce complications and shorten hospital stay. Discharge planning and follow-up require individualized pathways addressing nutritional status, body weight and muscle mass changes, anastomotic function, and long-term quality of life assessment and education (61).

### 5.6 Innovations in biodegradable stents and biomaterials

Stent technology, as an important means of maintaining pancreatic duct patency and promoting tissue healing during PJ, has garnered increasing attention. Traditional plastic or metal stents, while providing initial support, present long-term issues including foreign body reactions, infection, and restenosis. Recent advances in medicine-engineering integration have propelled research into novel materials such as biodegradable polymers and magnesium alloys. For instance, the ARCHIMEDES stent, fabricated from composite biodegradable materials with graded degradation rates, achieves integrated pancreatic juice drainage and support functions, with preliminary clinical data suggesting a potential advantage in reducing POPF rates. However, existing trials are all non-randomized, unblinded, and non-controlled, carrying a moderate risk of bias. Therefore, current evidence is insufficient to support routine clinical use of biodegradable stents, and high-quality randomized clinical trials comparing stent placement with no-stent controls are urgently needed to objectively assess their efficacy and safety (62).

Beyond mechanical properties and degradation profiles, conferring bioactive functions has become a key direction in biomaterials research related to PJ. The PEG/ExoM2 hydrogel, developed by Brightech in collaboration with Sichuan Provincial People’s Hospital experts, incorporates M2 macrophage-derived exosomes, exhibiting favorable anti-inflammatory properties while promoting angiogenesis and cell migration, thereby significantly improving healing quality in a porcine PJ model. However, the efficacy and safety of this hydrogel have only been preliminarily validated in animal models, and its value in human application awaits further confirmation by clinical trials (63). This material development not only signals the evolution of pancreatic duct stent technology from simple mechanical support toward integrated tissue repair, but also provides novel therapeutic approaches for the management of complications such as pancreatic fistula (**Table 3**).

**Table 3:** Emerging technologies for pancreaticojejunostomy.

Technology category	Specific technique/material	Advantages	Study type	Clinical readiness (whether suitable for routine use)
Biodegradable stent	ARCHIMEDES stent	Graded degradation	Prospective, non-randomized clinical study	Research use only; not recommended for routine clinical application.
Bioactive hydrogel	PEG/ExoM2 hydrogel	Anti-inflammatory, pro-angiogenic, pro-cell migration	Animal model	Still in animal or in vitro experimental stage.

## 6 Conclusion

As the core component of PD, PJ is continuously evolving toward greater precision and individualization. Current evidence indicates that PJ and PG have comparable overall POPF rates, with each technique offering distinct advantages. Furthermore, key techniques such as duct-to-mucosa anastomosis and invagination anastomosis can significantly reduce the risk of clinically relevant POPF (CR-POPF), while the selection of a specific anastomotic technique should be based on pancreatic texture, main pancreatic duct diameter, and surgeon experience. More importantly, systematic perioperative comprehensive management strategies—including preoperative nutritional support and prehabilitation, intraoperative individualized selection of anastomotic techniques based on pancreatic characteristics, postoperative drain monitoring and early removal, appropriate use of somatostatin analogues, early enteral nutrition, and implementation of ERAS pathways—have been demonstrated to significantly reduce POPF rates and improve patient outcomes.

Nevertheless, evidence in the following areas remains insufficient and subject to considerable uncertainty: the exact advantages of robot-assisted surgery and laparoscopic surgery in terms of PJ quality require further prospective randomized controlled trials for validation; the clinical value of indocyanine green (ICG) fluorescence perfusion imaging for assessing pancreatic remnant blood supply needs confirmation through multicenter, large-sample studies; and the efficacy and safety of novel materials such as biodegradable stents and bioactive hydrogels have only been derived from basic research and are not yet sufficient to support routine clinical application.

Future research should focus on the following directions: First, conduct high-quality, multicenter randomized controlled trials to compare different anastomotic techniques in high-risk patients (e.g., soft pancreas, small pancreatic ducts). Second, promote rigorous evaluation of biodegradable stents and bioactive materials transitioning from animal experiments to clinical application, including long-term patency rates and complications. Third, establish standardized perioperative comprehensive management pathways to validate their impact on the incidence and severity of POPF. Fourth, explore imaging-based radiomics and artificial intelligence-driven preoperative prediction models to achieve truly individualized anastomotic strategies. Fifth, perform long-term follow-up studies to systematically assess the impact of different anastomotic techniques on patients’ long-term quality of life, exocrine pancreatic function, and oncological outcomes.

In summary, the precision-oriented development of PJ is a multidimensional, interdisciplinary systematic endeavor. Through continuous technological innovation, rigorous clinical research, and systematic perioperative management, the safety and efficacy of PJ are expected to be further enhanced, ultimately delivering improved clinical outcomes and quality of life for patients undergoing pancreatic surgery.
